# Arterial stiffness is highly correlated with the scores obtained from the Steno Type 1 Risk Engine in subjects with T1DM

**DOI:** 10.1371/journal.pone.0220206

**Published:** 2019-09-04

**Authors:** Gemma Llauradó, Albert Cano, Lara Albert, Silvia Ballesta, Isabel Mazarico, María-Florencia Luchtenberg, Montserrat González-Sastre, Ana Megía, Rafael Simó, Joan Vendrell, José-Miguel González-Clemente

**Affiliations:** 1 Department of Endocrinology and Nutrition, Hospital del Mar, Institut Hospital del Mar d'Investigacions Mèdiques (IMIM), Barcelona, Spain; 2 IISPV Pere Virgili Health Research Institute, Tarragona, Spain; 3 Centro de Investigación Biomédica en Red de Diabetes y Enfermedades Metabólicas Asociadas (CIBERDEM), Instituto de Salud Carlos III, Madrid, Spain; 4 Department of Endocrinology and Nutrition, Parc Taulí Hospital Universitari, Institut d’Investigació i Innovació Parc Taulí I3PT, Universitat Autònoma de Barcelona, Sabadell, Spain; 5 Ophthalmology Department, Parc Taulí Hospital Universitari, Institut d’Investigació i Innovació Parc Taulí I3PT, Universitat Autònoma de Barcelona, Sabadell, Spain; 6 Endocrinology and Nutrition Section, Joan XXIII University Hospital, Rovira i Virgili University, Tarragona, Spain; 7 Diabetes and Metabolism Research Unit, Institut de Recerca Hospital Universitari Vall d'Hebron, Universitat Autònoma de Barcelona, Barcelona, Spain; University of Colorado Denver School of Medicine, UNITED STATES

## Abstract

**Objectives:**

Currently used risk scores for type 2 diabetes mellitus (T2DM) clearly underestimate cardiovascular risk in type 1 diabetes (T1DM). Hence, there is a need to develop novel and specific risk-estimation tools for this population. We aimed to assess the relationship between the Steno Type 1 Risk Engine (ST1RE) and arterial stiffness (AS), and to identify potential cut-off points of interest in clinical practice.

**Design and methods:**

A total of 179 patients with T1DM (50.8% men, mean age 41.2±13.1 years), without established cardiovascular disease, were evaluated for clinical and anthropometric data (including classical cardiovascular risk factors), and AS measured by aortic pulse-wave velocity (aPWV). The ST1RE was used to estimate 10-year cardiovascular risk and patients were classified into 3 groups: low- (<10%; n = 105), moderate- (10–20%; n = 53) and high-risk (≥20%; n = 21).

**Results:**

When compared with the low- and moderate-risk groups, patients in the high-risk group were older, had higher prevalence of hypertension, dyslipidemia and insulin-resistance, and had higher body-mass index and HbA_1c_. aPWV increased in parallel with estimated cardiovascular risk (6.4±1.0, 8.4±1.3 and 10.3±2.6m/s; p<0.001). As an evaluation of model performance, the C-statistic of aPWV was 0.914 (95% confidence interval [CI]:0.873–0.950) for predicting moderate/high-risk and 0.879 (95%CI:0.809–0.948) for high-risk, according to the ST1RE. The best cut-off points of aPWV were 7.3m/s (sensitivity:86%, specificity:83%) and 8.7m/s (sensitivity:76%, specificity:86%) for moderate/high- and high-risk, respectively.

**Conclusions:**

AS is highly correlated with the scores obtained from the ST1RE. We have identified two cut-off points of AS that can clearly discriminate moderate/high- and high-risk T1DM patients, which could be of great value in clinical practice.

## Introduction

Cardiovascular disease (CVD) is the leading cause of death in patients with type 1 diabetes mellitus (T1DM) [1], with coronary artery disease (CAD) as the principal manifestation [2]. Indeed, the relative risk of death from CAD in T1DM can be ten times greater than that in the non-diabetic population, especially in women, and it is even greater than the relative risk in type 2 diabetes (T2DM) [1, 3]. Moreover, T1DM causes a life expectancy loss of about 11 years for men and 13 years for women, with the largest percentage of the estimated loss in life expectancy related to ischemic heart disease (up to one-third) [4].

Arterial stiffness is an early indicator of arteriosclerosis and vascular damage [5] and, accordingly, it´s analysis could provide insights into arteriosclerotic mechanisms long before any cardiovascular event occurs. Arterial stiffness predicts cardiovascular events independently of classical cardiovascular risk factors in several populations [6–8], and its estimation has been demonstrated to improve cardiovascular risk prediction beyond the Framingham Risk Score [9, 10]. However, prospective studies assessing the prognostic value of aortic pulse wave velocity (aPWV), a marker of arterial stiffness, in subjects with T1DM are lacking.

Several risk scores have been developed to predict CVD both in the general population and specifically in patients with T2DM. However, neither general (Framingham Risk Score) nor T2DM (UKPDS Risk Engine) risk algorithms are sufficiently robust for risk prediction in T1DM [11, 12]. Accordingly, the Scientific Statement from the American Heart Association and the American Diabetes Association on CVD points to the need for novel risk-estimation tools for better prediction of cardiovascular events in T1DM and suggests the use of models specifically obtained from T1DM cohorts [2], such as the Steno Type 1 Risk Engine (ST1RE).

Against this background, the aims of the present study were to assess the relationship between the ST1RE and preclinical atherosclerosis measured as arterial stiffness, and to identify potential cut-off points of interest in clinical practice.

## Material and methods

### Study subjects

One hundred and seventy-nine patients aged 18–65 years, with T1DM and without established CVD were included in the study. Patients were consecutively recruited from our outpatient clinic from November 2008 to February 2014. T1DM was defined as an onset of diabetes before the age of 36 years and undetectable fasting levels of serum C-peptide (<0.6 ng/mL). Exclusion criteria included the following: i) presence of previous clinical CVD (CAD, cerebrovascular disease or peripheral artery disease) based on clinical registers, previous personal history of CVD and/or the presence of suggestive symptoms, ii) abnormal resting ECG, iii) any other acute/chronic condition associated with an inflammatory response (e.g., acute or chronic inflammatory or infectious diseases), iv) use of anti-inflammatory drugs in the previous 6 months, v) malignant disease in the previous 5 years (except basal cell carcinoma), vi) hospitalization in the previous 2 months, vii) arrhythmia (other than atrial premature complex), and viii) pregnancy. The study protocol was approved by our hospital ethics committee (Parc Taulí Research Ethics Committee) and was conducted in accordance with the Declaration of Helsinki. All subjects gave their written informed consent before participating in the study.

### Study design

All subjects underwent standard anamnesis and physical examination, as previously described [13, 14]. The following information was recorded using a predefined standardized form: age, sex, diabetes duration, family history of premature CVD (defined as CVD occurring before the age of 55 in males and 65 in female first-degree relatives), physical activity (International Physical Activity Questionnaire) [15], active smoking, alcohol intake, insulin dose, and the use of any other medication. Body weight, height, and waist and hip circumference were registered. Systolic and diastolic blood pressure (SBP and DBP, respectively) were measured and mean arterial pressure (MAP) was calculated as 1/3 SBP + 2/3 DBP. Venous blood samples were taken after an overnight fast and complete blood counts, fasting plasma glucose, HbA_1c_, creatinine and lipid profile were determined. Hypertension was defined as BP >140/90 [16] and/or taking antihypertensive drugs. Dyslipidemia was defined as having concentrations of total cholesterol >5.2mmol/L, triglycerides >1.7 mmol/L, HDL-cholesterol <1.03mmol/L, LDL- cholesterol >3.4mmol/L [17] and/or receiving drug treatment for dyslipidemia.

### Laboratory analyses

HbA_1c_ was determined by high-performance liquid chromatography (Menarini Diagnostics, Firenze, Italy). Total serum cholesterol, triglycerides and HDL-cholesterol were measured using standard enzymatic methods. LDL-cholesterol was estimated with the Friedewald formula [18].

### Insulin resistance

To estimate insulin resistance, we used the formula proposed by Williams et al. for patients with T1DM [19], subsequently adapted for the use of HbA_1c_ rather than HbA_1_ by Kilpatrick et al. for its use in the DCCT/EDIC cohort [20]. It yields an estimate of the glucose disposal rate (eGDR), taking into account glycemic control, waist-to-hip ratio (WHR) and blood pressure (eGDR = 24.31–12.22 × [WHR] - 3.29 × [hypertension 0 = No; 1 = Yes] - 0.57 × [HbA_1c_]) [20]. The formula was validated against euglycemic-hyperinsulinemic clamp in a group of patients with T1DM clinically similar to those evaluated in the present study. Lower eGDR values reflect higher insulin resistance levels.

### Assessment of microvascular complications

Peripheral polyneuropathy was assessed through a previously described two-step protocol combining the 15-item MNSI questionnaire and a physical examination [21]. The same ophthalmologist evaluated the presence and degree of diabetic retinopathy in all patients, who were then classified into the following three groups according to the degree of retinopathy: no retinopathy, non-proliferative retinopathy, or proliferative retinopathy. Nephropathy was assessed by the measurement of the urinary albumin/creatinine ratio. Subjects with a ratio greater than 3.4 mg/mmol [22], or previously treated with converting enzyme inhibitors or angiotensin receptor blockers (for microalbuminuria or macroalbuminuria), were classified as having diabetic nephropathy.

### Measurement of arterial stiffness

We measured aPWV, a gold standard for measuring arterial stiffness, as recommended by current international consensus [23]. The method has been previously described in detail [14]. Briefly, aPWV was determined by sequential applanation tonometry using a Millar tonometer (SPC-301, Millar Instruments, Houston, TX, USA) at the carotid and femoral arteries, gated to three-lead electrocardiography using the SphygmoCor system (AtCor Medical Pty Ltd., West Ryde (Sydney), NSW, Australia). Those aPWV recordings not satisfying the automatic quality controls specified by the SphygmoCor software were rejected. The mean of two aPWV measurements was used for each subject for all calculations. Data were available for all the participants included in the study.

### Steno Type 1 Risk Engine

Our data were used to calculate the 10-year probability of CVD according to the ST1RE [12]. This score considers the following variables: age, gender, smoking habit, exercise, T1DM duration, systolic blood pressure, LDL-cholesterol, HbA_1c_, renal function and micro-/macroalbuminuria. Based on the score obtained, patients were classified into 3 groups: low- (<10%; n = 105), moderate- (10–20%; n = 53) and high-risk (≥20%; n = 21).

### Statistical analyses

All data were tested for normality using the Shapiro-Wilk test. Data are presented as percentage, mean (standard deviation) for normally distributed quantitative variables, or median (interquartile range) for non-normally distributed quantitative variables. One-way analysis of variance (ANOVA) or the Kruskal-Wallis test was used for comparisons between groups of normally and non-normally distributed quantitative variables, as needed. The Bonferroni procedure (parametric) and the Dunn's test (non-parametric) were used for *post hoc* analyses for multiple comparisons. We tested aPWV discrimination using the C-statistic derived from the logistic regression models. Receiver-operating characteristic (ROC) curves were developed to represent the prediction of 10-year probability of CVD risk (based on the equation obtained from ST1RE), in which sensitivity is plotted as a function of 1-specificity, for both moderate/high- and high-risk groups. The best aPWV cut-off point was selected based on the Youden index calculation. Two-tailed p-values <0.05 were considered statistically significant. All calculations were made using STATA v.13.1 for Mac (StataCorp LP, College Station, TX, USA).

## Results

**Fig 1:**
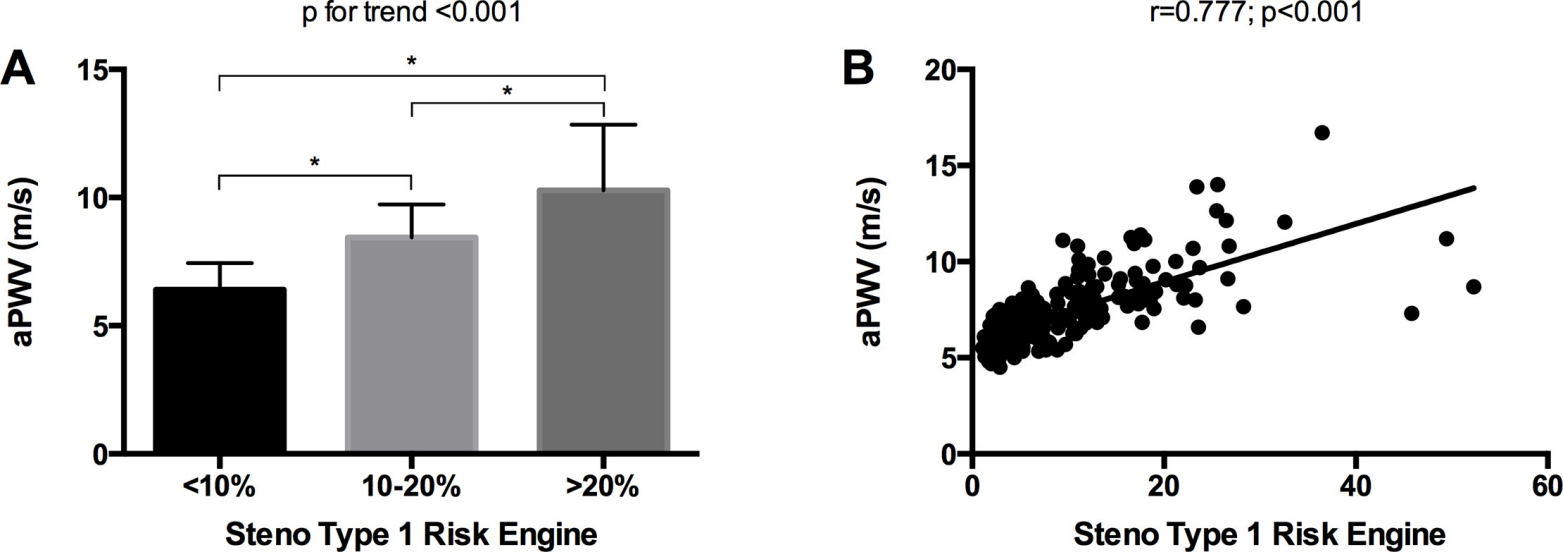
A. Comparison of aortic pulse wave velocity (aPWV) for the Steno Type 1 Risk Engine (ST1RE) low- (<10%), moderate- (10–20%) and high-risk (≥20%) groups (*p<0.05 for multiple comparison). B. Spearman correlation coefficient for the association between aPWV and ST1RE risk score.

A total of 179 patients with T1DM were included. The main clinical and analytical characteristics of the study population, stratified by CVD risk (low, moderate and high), are shown in Table 1. When compared with the low- and moderate-risk groups, patients in the high-risk group were older and had a higher prevalence of hypertension and dyslipidemia. They also had a higher body-mass index (BMI), higher insulin resistance, worse glycemic control and higher prevalence of microvascular complications. aPWV increased in parallel with estimated cardiovascular risk (Fig 1, panel A).

**Table 1:** Clinical and metabolic characteristics of patients with type 1 diabetes stratified by 10-year CVD risk according to the Steno Type 1 Risk Engine.

	Whole population (n = 179)	Low-risk (n = 105)	Moderate-risk (n = 53)	High-risk (n = 21)	p for trend
***Clinical characteristics***					
Age (yrs.)	41.2 (13.1)	32.5 (8.3)	50.8 (6.0)*	60.7 (6.6)^†^^,^^‡^	<0.001
Gender (male/female), n	91/88	52/53	29/24	10/11	NS
Current smokers, n (%)	57 (31.8)	31.0 (29.5)	21 (39.6) *	5 (23.8)	0.012
Family history of premature CVD, n (%)	16 (8.9)	7 (6.7)	6 (11.3)	3 (14.3)	NS
Family history of T2DM, n (%)	37 (20.7)	16 (15.2)	15 (28.3)	6 (28.6)	NS
Hypertension, n (%)	49 (27.4)	15 (14.3)	20 (33.7) *	14 (66.7)^†^	<0.001
Dyslipidemia, n (%)	76 (58.5)	24 (36.4)	35 (77.8) *	17 (89.5)^†^	<0.001
***Diabetes***					
Diabetes duration (yrs.)	16 (12–23)	14 (20–22)	18 (15–27) *	20 (15–29)^†^	<0.001
Total insulin doses (UI/kg·day)	0.6 (0.5–0.8)	0.6 (0.5–0.8)	0.7 (0.6–0.8)	0.6 (0.5–0.7)	NS
Microvascular complications, n (%)	68 (38.4)	28 (27.2)	23 (43.4)	18 (81.0) ^†^^,^^‡^	<0.001
Retinopathy, n (%)					NS
None	138 (77.1)	86 (81.9)	40 (75.5)	12 (57.1)	
Non-proliferative	20 (11.2)	9 (8.6)	6 (11.3)	5 (23.8)	
Proliferative	21 (11.7)	10 (9.5)	7 (13.2)	4 (19.1)	
Nephropathy, n (%)	41 (23.2)	14 (13.6)	15 (28.3)	4 (19.1)^†^	<0.001
Peripheral neuropathy, n (%)	7 (3.9)	1 (1.0)	3 (5.7)	3 (14.3)^†^	0.011
***Anthropometric measurements***					
Weight (kg)	71.7 (13.0)	69.8 (12.4)	75.2 (14.3)*	72.0 (10.7)	0.045
BMI (kg/m^2^)	25.4 (3.7)	24.3 (3.2)	26.6 (3.8)*	27.8 (4.4)^†^	<0.001
Waist-to-hip ratio	0.88 (0.81–0.94)	0.84 (0.77–0.90)	0.93 (0.86–0.99) *	0.94 (0.90–0.98)^†^	<0.001
***Blood pressure***					
SBP (mmHg)	125.6 (12.1)	121.8(11.0)	128.8(11.2)*	136.9(10.7)^†^^,^^‡^	<0.001
DBP (mmHg)	72.0 (8.9)	70.1 (8.2)	74.4 (8.7)*	75.7 (10.1)^†^	0.002
MAP (mmHg)	89.9 (9.1)	87.3 (8.4)	92.5 (8.5)	96.1 (9.6)	<0.001
***Laboratory parameters***					
Fasting plasma glucose (mmol/L)	8.2 (3.8)	7.8 (3.5)	8.4 (3.8)	9.5 (4.5)	NS
HbA_1c_ (%)	7.8 (1.0)	7.6 (1.0)	8.0 (1.0)	8.5 (1.1)^†^	<0.001
HbA_1c_ (mmol/mol)	61.8 (11.4)	59.2 (11.0)	63.7 (10.5)	69.9 (11.6)	
Urinary ACR (mg/g)	4.7 (2.7–10.6)	4.1 (2.4–7.7)	6.1 (3.0–9.8)	14.0 (5.3–54.0)^†^^,^^‡^	<0.001
Total cholesterol (mmol/L)	4.6 (4.1–5.2)	4.5 (4.0–5.1)	4.7 (4.3–5.2)	5.1 (4.4–5.8)	NS
HDL-cholesterol (mmol/L)	1.7 (1.3–2.0)	1.6 (1.3–1.9)	1.7 (1.4–2.1)	1.7 (1.5–2.2)	0.073
LDL-cholesterol (mmol/L)	2.5 (2.1–2.9)	2.5 (2.2–2.9)	2.4 (2.1–2.8)	2.6 (2.3–3.1)	NS
Triglycerides (mmol/L)	0.73 (0.60–0.90)	0.72 (0.54–0.88)	0.73 (0.63–0.90)	0.76 (0.68–1.1)^†^	NS
***Insulin resistance***					
eGDR (mg/kg/min)	8.6 (6.1–10.0)	9.5 (8.4–10.4)	6.9 (5.4–8.8)*	5.6 (4.1–6.8)^†^^,^^‡^	<0.001
***Arterial stiffness***					
aPWV (m/s)	7.5 (1.9)	6.4 (1.0)	8.4 (1.3)*	10.3 (2.5)^†^^,^^‡^	<0.001

Data are given as percentages, mean (SD) or median (interquartile range). CVD: cardiovascular disease. T2DM: type 2 diabetes. BMI: body mass index. WHR: waist-to-hip ratio. SBP: systolic blood pressure. DBP: diastolic blood pressure. MAP: mean arterial pressure. ACR: urinary albumin to creatinine ratio. eGDR: estimation of glucose disposal rate. aPWV: aortic pulse wave velocity.

*p<0.05 for moderate-risk compared with low-risk

^†^p < .0.05 for high-risk compared with low-risk and

^‡^p<0.05 for high-risk compared with moderate-risk.

In univariate analyses, age (r = 0.941; p<0.001), smoking habit (r = 0.228; p = 0.006), family history of T2DM (r = 0.184; p = 0.014), arterial hypertension (r = 0.415; p<0.001), SBP (r = 0.439; p<0.001), DBP (r = 0.334; p<0.001) and MAP (r = 0.415; p<0.001), dyslipidemia (r = 0.518; p<0.001), total cholesterol (r = 0.187; p = 0.033), diabetes duration (r = 0.274; p<0.001), chronic complications (r = 0.359; p<0.001), diabetic nephropathy (r = 0.360; p<0.001), peripheral neuropathy (r = 0.201; p = 0.007), BMI (r = 0.307; p<0.001), WHR (r = 0.563; p<0.001), HbA_1c_ (r = 0.252; p<0.001) and aPWV (r = 0.777; p<0.001) were positively associated with the ST1RE score (Fig 1, panel B).

**Fig 2:**
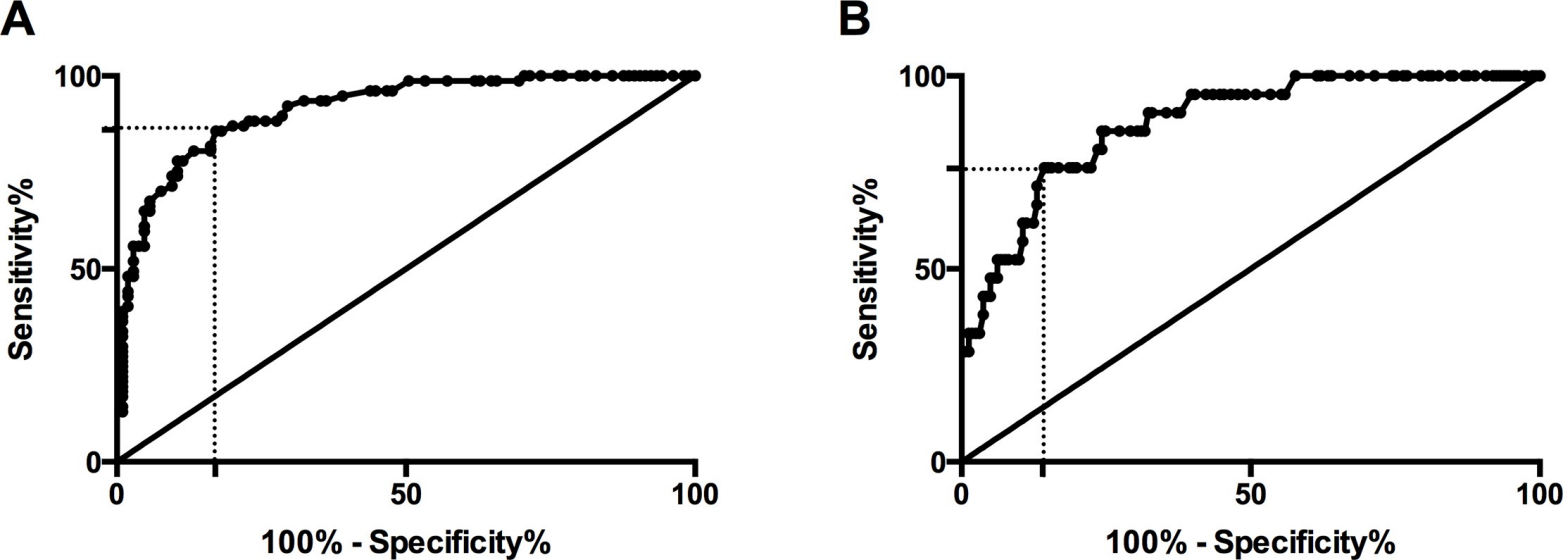
ROC curves for aPWV to identify cardiovascular risk according to the ST1RE for moderate/high- (panel A) and high-risk groups (panel B).

To evaluate the potential role of aPWV for predicting cardiovascular risk according to the ST1RE, we developed one regression model for moderate/high-risk patients and another model for high-risk patients. aPWV was positively associated both with moderate/high- and high-risk according to the ST1RE, with an odds ratio (OR) of 4.73 (95% confidence interval [CI] 3.00–7.45; p<0.001) and 2.28 (95%CI: 1.66–3.12; p<0.001), respectively. The C-statistic of aPWV was 0.914 (95%CI: 0.873–0.95) for predicting moderate/high-risk and 0.879 (95%CI: 0.809–0.948) for high-risk according to the ST1RE (Fig 2). The best cut-off points of aPWV were 7.3 m/s (sensitivity: 86% and specificity: 83%) and 8.7 m/s (sensitivity: 76% and specificity: 86%) for moderate/high and high-risk, respectively.

## Discussion

The main finding of the present study is that arterial stiffness is highly correlated with the scores obtained from the ST1RE in patients with T1DM and without previous CVD. In addition, we have identified two cut-off points of arterial stiffness that can clearly discriminate moderate/high- and high-risk T1DM patients, which could be of significant value in clinical practice.

aPWV predicts cardiovascular events and total and cardiovascular mortality in the general population, in the elderly, and in patients with hypertension, end-stage renal failure or T2DM [6]. The independent predictive value of arterial stiffness has been proven after adjustment for classical cardiovascular risk factors and its estimation has also been demonstrated to improve cardiovascular risk prediction beyond the Framingham Risk Score [9, 10]. This suggests that measurement of arterial stiffness could add value to the classical cardiovascular risk factors in the prediction of cardiovascular risk. A possible explanation for this is that arterial stiffness integrates the damage of cardiovascular risk factors (known and unknown) on the aortic wall since birth, whereas classical cardiovascular risk factors can fluctuate over time, and hence their value at any given time may not reflect their real impact on arterial wall damage over the lifetime [23].

It has been reported that the currently used risk scores (designed for general or T2DM populations) clearly underestimate cardiovascular risk in the T1DM population [11, 12]. Accordingly, it is of special interest to develop specific cardiovascular risk models for these patients [2].

Three different risk scores have been recently developed for predicting CVD in T1DM, which differ in several aspects such as the number (between 4 and 11) and type of variables employed, and the population used for developing the models. However, none of them have been used thus far in routine clinical practice [2, 24]. The first risk score was developed in a childhood-onset T1DM cohort [25]; the second one, based on the Swedish National Diabetes Register [26], included patients with previous cardiovascular events (including patients in primary and secondary prevention), and the third one, the ST1RE [12], was developed and validated in two cohorts of adult patients with T1DM without established CVD (similar to our patient group).

A possible reason for the high correlation between arterial stiffness and the ST1RE score could be the similarities between the two cohorts in terms of age (41.2 years vs. 42.2 years), gender distribution (50.8% vs. 54.2% of males) and duration of T1DM (16.0 years vs. 16.6 years). Given our results and the fact that arterial stiffness has the potential to improve the value of classical cardiovascular risk factors for predicting CVD, we believe that its assessment could be of great value in clinical practice, improving the cardiovascular risk stratification and the follow-up of patients with T1DM without established CVD.

In this study, we found that the discrimination of aPWV was excellent with a C-statistic of 0.914 for predicting moderate/high-risk, and 0.879 for high-risk. In addition, we identified two potential cut-off points of arterial stiffness that can clearly discriminate moderate/high- (7.3m/s) and high-risk (8.7m/s) T1DM patients, which could be of significant value in clinical practice. Nevertheless, we acknowledge that one important limitation of aPWV is how distance is assessed. In our setting, we used the subtraction of suprasternal notch-to-carotid distance from the suprasternal notch-to-femoral distance, as previously described [14, 23]. Prior studies comparing the distance measured using magnetic resonance imaging (a direct measure of aortic stiffness) have shown that the subtraction method underestimates the distance measurements and overestimates aPWV. For this reason, some authors have recently proposed new distance measurements that would improve the estimation of the carotid-femoral path length [27]. Of all currently used distances, the 80% of the direct carotid-femoral distance (common carotid artery—common femoral artery × 0.8) has been proposed as the most accurate. Consequently, a new standard cut-off value aPWV of 10m/s has been proposed as indicative of subclinical organ damage and increased risk for cardiovascular events [28], instead of the previously established cut-off value of 12m/s proposed in 2007 [29]. Thus, it should be acknowledged that the method used to measure the distance needs to be taken into consideration when interpreting our results, as it can clearly modify our proposed cut-off points derived from the ROC curves.

We also acknowledge that prospective studies are needed to investigate the potential role of arterial stiffness for predicting cardiovascular events in subjects with T1DM. To the best of our knowledge, only one such study has attempted to evaluate the relationship between central arterial stiffness and the prediction of cardiovascular events in T1DM. This study showed that central pulse pressure as a measure of central arterial stiffness was more strongly associated with the prediction of cardiovascular events than the augmentation index (an indirect measure of arterial stiffness), but no data on aPWV were reported [30]. We are aware that estimated 10-year CVD risk is an imperfect end-point to test the potential predictive role of aPWV, as it does not represent true disease status and there is the possibility to misclassify patients into the wrong risk group (as in all algorithms designed to predict cardiovascular risk). Also, the validation of ST1RE was performed for the 5-year data and most participants were Danish. Thus, additional studies exploring the potential role of arterial stiffness to predict future events and validated in other populations are needed.

The major limitation of the current study is its cross-sectional design, which makes it impossible to determine the temporal ordering of the association between arterial stiffness and cardiovascular events. In addition, the young age and the small sample size (especially in the high-risk group) could underestimate our results. Nonetheless, it seems reasonable to believe that this score might add predictive value to the classical cardiovascular risk models. In addition, the study was observational in design and consequently complete control of all potential (unknown) confounding factors could not be ensured.

## Conclusions

In summary, our study demonstrates that aPWV is highly correlated with the scores obtained from the ST1RE in a Mediterranean cohort of subjects with T1DM without previous CVD. We have identified two cut-off points of arterial stiffness that can clearly discriminate moderate/high- and high-risk patients. These cut-offs could be of great interest in clinical practice. Our findings suggest that measurement of arterial stiffness is a useful tool for detecting subclinical arteriosclerosis and contributes to better cardiovascular prediction in T1DM. Nevertheless, prospective studies are needed to clarify the relationship between arterial stiffness and macrovascular complications in subjects with T1DM.

## Supporting information

S1 File DATA SET. (DTA)

## References

[1] Libby P, Nathan DM, Abraham K, Brunzell JD, Fradkin JE, Haffner SM, et al Report of the National Heart, Lung, and Blood Institute-National Institute of Diabetes and Digestive and Kidney Diseases Working Group on Cardiovascular Complications of Type 1 Diabetes Mellitus. Circulation. 2005;111(25):3489–93. 10.1161/CIRCULATIONAHA.104.529651 . 15983263

[2] de Ferranti SD, de Boer IH, Fonseca V, Fox CS, Golden SH, Lavie CJ, et al Type 1 diabetes mellitus and cardiovascular disease: a scientific statement from the American Heart Association and American Diabetes Association. Diabetes care. 2014;37(10):2843–63. Epub 2014/08/13. 10.2337/dc14-1720 25114297 PMC4170130

[3] Orchard TJ, Costacou T, Kretowski A, Nesto RW. Type 1 diabetes and coronary artery disease. Diabetes care. 2006;29(11):2528–38. 10.2337/dc06-1161 . 17065698

[4] Livingstone SJ, Levin D, Looker HC, Lindsay RS, Wild SH, Joss N, et al Estimated life expectancy in a Scottish cohort with type 1 diabetes, 2008–2010. JAMA. 2015;313(1):37–44. 10.1001/jama.2014.16425 25562264 PMC4426486

[5] Cavalcante JL, Lima JA, Redheuil A, Al-Mallah MH. Aortic stiffness: current understanding and future directions. Journal of the American College of Cardiology. 2011;57(14):1511–22. 10.1016/j.jacc.2010.12.017 . 21453829

[6] Vlachopoulos C, Aznaouridis K, Stefanadis C. Prediction of cardiovascular events and all-cause mortality with arterial stiffness: a systematic review and meta-analysis. Journal of the American College of Cardiology. 2010;55(13):1318–27. 10.1016/j.jacc.2009.10.061 . 20338492

[7] Ben-Shlomo Y, Spears M, Boustred C, May M, Anderson SG, Benjamin EJ, et al Aortic pulse wave velocity improves cardiovascular event prediction: an individual participant meta-analysis of prospective observational data from 17,635 subjects. Journal of the American College of Cardiology. 2014;63(7):636–46. 10.1016/j.jacc.2013.09.063 24239664 PMC4401072

[8] Zhong Q, Hu MJ, Cui YJ, Liang L, Zhou MM, Yang YW, et al Carotid-Femoral Pulse Wave Velocity in the Prediction of Cardiovascular Events and Mortality: An Updated Systematic Review and Meta-Analysis. Angiology. 2017:3319717742544 10.1177/0003319717742544 . 29172654

[9] Mitchell GF, Hwang SJ, Vasan RS, Larson MG, Pencina MJ, Hamburg NM, et al Arterial stiffness and cardiovascular events: the Framingham Heart Study. Circulation. 2010;121(4):505–11. Epub 2010/01/20. 10.1161/CIRCULATIONAHA.109.886655 20083680 PMC2836717

[10] van Sloten TT, Sedaghat S, Laurent S, London GM, Pannier B, Ikram MA, et al Carotid stiffness is associated with incident stroke: a systematic review and individual participant data meta-analysis. Journal of the American College of Cardiology. 2015;66(19):2116–25. 10.1016/j.jacc.2015.08.888 . 26541923

[11] Zgibor JC, Piatt GA, Ruppert K, Orchard TJ, Roberts MS. Deficiencies of cardiovascular risk prediction models for type 1 diabetes. Diabetes care. 2006;29(8):1860–5. Epub 2006/07/29. 10.2337/dc06-0290 . 16873793

[12] Vistisen D, Andersen GS, Hansen CS, Hulman A, Henriksen JE, Bech-Nielsen H, et al Prediction of First Cardiovascular Disease Event in Type 1 Diabetes Mellitus: The Steno Type 1 Risk Engine. Circulation. 2016;133(11):1058–66. 10.1161/CIRCULATIONAHA.115.018844 . 26888765

[13] Llaurado G, Cano A, Hernandez C, Gonzalez-Sastre M, Rodriguez AA, Punti J, et al Type 1 diabetes: Developing the first risk-estimation model for predicting silent myocardial ischemia. The potential role of insulin resistance. PLoS One. 2017;12(4):e0174640 10.1371/journal.pone.0174640 28369151 PMC5378337

[14] Llaurado G, Ceperuelo-Mallafre V, Vilardell C, Simo R, Freixenet N, Vendrell J, et al Arterial stiffness is increased in patients with type 1 diabetes without cardiovascular disease: a potential role of low-grade inflammation. Diabetes care. 2012;35(5):1083–9. Epub 2012/02/24. 10.2337/dc11-1475 22357186 PMC3329819

[15] Hallal PC, Victora CG. Reliability and validity of the International Physical Activity Questionnaire (IPAQ). Medicine and science in sports and exercise. 2004;36(3):556 . 15076800 10.1249/01.mss.0000117161.66394.07

[16] Williams B, Mancia G, Spiering W, Agabiti Rosei E, Azizi M, Burnier M, et al 2018 ESC/ESH Guidelines for the management of arterial hypertension. European heart journal. 2018;39(33):3021–104. 10.1093/eurheartj/ehy339 . 30165516

[17] National Cholesterol Education Program Expert Panel on Detection E, Treatment of High Blood Cholesterol in A. Third Report of the National Cholesterol Education Program (NCEP) Expert Panel on Detection, Evaluation, and Treatment of High Blood Cholesterol in Adults (Adult Treatment Panel III) final report. Circulation. 2002;106(25):3143–421. Epub 2002/12/18. . 12485966

[18] Friedewald WT, Levy RI, Fredrickson DS. Estimation of the concentration of low-density lipoprotein cholesterol in plasma, without use of the preparative ultracentrifuge. Clinical chemistry. 1972;18(6):499–502. . 4337382

[19] Williams KV, Erbey JR, Becker D, Arslanian S, Orchard TJ. Can clinical factors estimate insulin resistance in type 1 diabetes? Diabetes. 2000;49(4):626–32. 10.2337/diabetes.49.4.626 . 10871201

[20] Kilpatrick ES, Rigby AS, Atkin SL. Insulin resistance, the metabolic syndrome, and complication risk in type 1 diabetes: "double diabetes" in the Diabetes Control and Complications Trial. Diabetes care. 2007;30(3):707–12. 10.2337/dc06-1982 . 17327345

[21] Gonzalez-Clemente JM, Gimenez-Perez G, Richart C, Broch M, Caixas A, Megia A, et al The tumour necrosis factor (TNF)-alpha system is activated in accordance with pulse pressure in normotensive subjects with type 1 diabetes mellitus. European journal of endocrinology / European Federation of Endocrine Societies. 2005;153(5):687–91. 10.1530/eje.1.02016 . 16260427

[22] American Diabetes A. Standards of medical care in diabetes—2011. Diabetes care. 2011;34 Suppl 1:S11–61. Epub 2011/01/14. 10.2337/dc11-S011 21193625 PMC3006050

[23] Laurent S, Cockcroft J, Van Bortel L, Boutouyrie P, Giannattasio C, Hayoz D, et al Expert consensus document on arterial stiffness: methodological issues and clinical applications. European heart journal. 2006;27(21):2588–605. 10.1093/eurheartj/ehl254 . 17000623

[24] American Diabetes A. 9. Cardiovascular Disease and Risk Management: Standards of Medical Care in Diabetes-2018. Diabetes care. 2018;41(Suppl 1):S86–S104. 10.2337/dc18-S009 . 29222380

[25] Zgibor JC, Ruppert K, Orchard TJ, Soedamah-Muthu SS, Fuller J, Chaturvedi N, et al Development of a coronary heart disease risk prediction model for type 1 diabetes: the Pittsburgh CHD in Type 1 Diabetes Risk Model. Diabetes research and clinical practice. 2010;88(3):314–21. 10.1016/j.diabres.2010.02.009 20236721 PMC2891292

[26] Cederholm J, Eeg-Olofsson K, Eliasson B, Zethelius B, Gudbjornsdottir S, Swedish National Diabetes R. A new model for 5-year risk of cardiovascular disease in Type 1 diabetes; from the Swedish National Diabetes Register (NDR). Diabet Med. 2011;28(10):1213–20. 10.1111/j.1464-5491.2011.03342.x . 21627687

[27] Huybrechts SA, Devos DG, Vermeersch SJ, Mahieu D, Achten E, de Backer TL, et al Carotid to femoral pulse wave velocity: a comparison of real travelled aortic path lengths determined by MRI and superficial measurements. Journal of hypertension. 2011;29(8):1577–82. Epub 2011/06/15. 10.1097/HJH.0b013e3283487841 . 21666491

[28] Van Bortel LM, Laurent S, Boutouyrie P, Chowienczyk P, Cruickshank JK, De Backer T, et al Expert consensus document on the measurement of aortic stiffness in daily practice using carotid-femoral pulse wave velocity. Journal of hypertension. 2012;30(3):445–8. Epub 2012/01/27. 10.1097/HJH.0b013e32834fa8b0 . 22278144

[29] Mancia G, De Backer G, Dominiczak A, Cifkova R, Fagard R, Germano G, et al 2007 Guidelines for the Management of Arterial Hypertension: The Task Force for the Management of Arterial Hypertension of the European Society of Hypertension (ESH) and of the European Society of Cardiology (ESC). Journal of hypertension. 2007;25(6):1105–87. 10.1097/HJH.0b013e3281fc975a . 17563527

[30] Gordin D, Waden J, Forsblom C, Thorn LM, Rosengard-Barlund M, Heikkila O, et al Arterial stiffness and vascular complications in patients with type 1 diabetes: The Finnish Diabetic Nephropathy (FinnDiane) Study. Annals of medicine. 2010 10.3109/07853890.2010.530681 . 21047152

